# Acyl-CoA thioesterase 7 is involved in cell cycle progression via regulation of PKC*ζ*–p53–p21 signaling pathway

**DOI:** 10.1038/cddis.2017.202

**Published:** 2017-05-18

**Authors:** Seung Hee Jung, Hyung Chul Lee, Hyun Jung Hwang, Hyun A Park, Young-Ah Moon, Bong Cho Kim, Hyeong Min Lee, Kwang Pyo Kim, Yong-Nyun Kim, Byung Lan Lee, Jae Cheol Lee, Young-Gyu Ko, Heon Joo Park, Jae-Seon Lee

**Affiliations:** 1 Department of Molecular Medicine, Inha University College of Medicine, Incheon, Korea; 2 Hypoxia-Related Disease Research Center, Inha University College of Medicine, Incheon, Korea; 3 Division of Basic Radiation Bioscience, Korea Institute of Radiological and Medical Sciences, Seoul, Korea; 4 Department of Applied Chemistry, College of Applied Science, Kyung Hee University, Yongin, Korea; 5 Division of Cancer Biology, Research Institute, National Cancer Center, Goyang, Korea; 6 Department of Anatomy, Seoul National University College of Medicine, Seoul, Korea; 7 Department of Oncology, Asan Medical Center, College of Medicine, University of Ulsan, Seoul, Korea; 8 Division of Life Sciences, Korea University, Seoul, Korea; 9 Department of Microbiology, Inha University College of Medicine, Incheon, Korea

## Abstract

Acyl-CoA thioesterase 7 (ACOT7) is a major isoform of the ACOT family that catalyzes hydrolysis of fatty acyl-CoAs to free fatty acids and CoA-SH. However, canonical and non-canonical functions of ACOT7 remain to be discovered. In this study, for the first time, ACOT7 was shown to be responsive to genotoxic stresses such as ionizing radiation (IR) and the anti-cancer drug doxorubicin in time- and dose-dependent manners. ACOT7 knockdown induced cytostasis via activation of the p53–p21 signaling pathway without a DNA damage response. PKC*ζ* was specifically involved in ACOT7 depletion-mediated cell cycle arrest as an upstream molecule of the p53–p21 signaling pathway in MCF7 human breast carcinoma and A549 human lung carcinoma cells. Of the other members of the ACOT family, including ACOT1, 4, 8, 9, 11, 12, and 13 that were expressed in human, ACOT4, 8, and 12 were responsive to genotoxic stresses. However, none of those had a role in cytostasis via activation of the PKC*ζ*–p53–p21 signaling pathway. Analysis of the ACOT7 prognostic value revealed that low ACOT7 levels prolonged overall survival periods in breast and lung cancer patients. Furthermore, ACOT7 mRNA levels were higher in lung cancer patient tissues compared to normal tissues. We also observed a synergistic effect of ACOT7 depletion in combination with either IR or doxorubicin on cell proliferation in breast and lung cancer cells. Together, our data suggest that a low level of ACOT7 may be involved, at least in part, in the prevention of human breast and lung cancer development via regulation of cell cycle progression.

Acyl-CoA thioesterases (ACOTs) are enzymes that catalyze hydrolysis of fatty acyl-CoAs to free fatty acids and CoA-SH. ACOTs are expressed ubiquitously in prokaryotes and eukaryotes.^1^ In higher organisms, different ACOT isoforms are localized to distinct cellular organelles, including peroxisomes, endoplasmic reticulum, cytosol and mitochondria. ACOTs have various chain length specificities on fatty acyl-CoAs and are distributed across a wide range of mammalian tissues.^2, 3, 4^ To date, 13 mammalian genes have been identified as ACOT superfamily members,^2, 3, 5^ and they are highly regulated by peroxisome proliferator-activated receptors (PPARs) and other nutritional factors.^1^ Although their physiological functions remain incompletely understood, recent gene knockout and in-depth characterization studies have revealed that a subgroup of ACOTs profoundly affect lipid metabolism and cellular signaling.^4, 6, 7, 8, 9^ ACOTs are broadly grouped into two classes based on enzyme molecular weight.^2^ Despite catalyzing the same reaction, these two classes are not structurally similar and do not share sequence homology.^3, 10^ Type 1 enzymes (ACOTs 1–6) are localized to the cytosol (ACOT1), mitochondria (ACOT2), and peroxisomes (ACOT3–ACOT6).^9, 11, 12, 13^ The larger (>100 kDa of molecular weight) oligomeric type II ACOTs (ACOTs 7–13) have different cellular localizations depending on the isoform.^9, 10, 14^

ACOT7 (also known as BACH, CTE-II, ACT, ACH1, and BACHa) is one of the most extensively studied ACOTs as a major isoform of the ACOT family.^9^ The enzyme is localized to the cytosol and is highly expressed in brain tissue and testis,^15, 16^ showing a preference for long-chain acyl-CoA substrates with fatty acid chains of 8–16 carbon atoms (C8–C16).^17, 18^ ACOT7 is primarily implicated in the hydrolysis of arachidonyl-CoA (AA-CoA) to arachidonic acid (AA) and CoA. AA is an important precursor molecule for proinflammatory eicosanoids and plays a number of important cellular roles in cell signaling, regulation of cell proliferation, and activation of metabolic enzymes.^9, 19, 20^ ACOT7 has recently been identified as playing a role in neurotoxic prevention through regulation of neuronal fatty acid metabolism.^21^ Forwood *et al.* demonstrated that ACOT7 is a candidate drug target in inflammatory disease, as overexpression of ACOT7 was shown to alter production of prostaglandins D2 and E2 in a macrophage cell line.^9, 22^ However, the roles of ACOT7 under various stressful conditions remain to be further revealed.

Protein kinase C (PKC) is involved in a variety of cellular functions, including cell proliferation, malignant proliferation, differentiation, and cell death.^23^ The PKC family is composed of at least 10 serine–threonine kinases based on their structural components and activation mechanism, and they are subdivided into three groups in mammals: classical or calcium-dependent (PKC*α*, *β*1, *β*2, and *γ*), novel or calcium-independent (PKC*δ*, *ε*, *η*, and *θ*), and atypical (PKC*ζ* and *λ*/ι). Classical PKCs are activated by oncogenic Kras or growth factors, leading to similar downstream signaling for control of cancer cell survival and metastasis.^24^ Among novel PKCs, whereas PKC*ε* and PKC*δ* have been implicated in cancer development or progression, relatively little is known about PKC*η* and PKC*θ*.^25, 26, 27, 28^ Atypical PKCs are structurally and functionally distinct from other PKCs. PKC*ζ* and PKC*λ*/ι as atypical PKCs have 84% amino-acid sequence homology in their kinase domains and contain a distinct N-terminal structural domain called Phox/Bem1 (PB1) that is specific for this subfamily.^29, 30^ PKC*ζ* and PKC*λ*/ι do not seem to be functionally redundant and cannot compensate for each other.^31^ All PKC isoforms share a highly conserved catalytic domain and more divergent regulatory domain.^32^ These relatively less conserved regulatory domains have different structures that affect the sensitivity of each PKC to various stimuli. PKCs seem to be involved in all aspects of tumorigenesis, including initiation, progression, and metastasis.^33^ The existence of various PKC isoforms suggests specialized roles for isoforms in the control of cellular functions.^34^ For example, whereas PKC*ε* induces cell proliferation, PKC*δ* inhibits growth. Further studies should elucidate the molecular mechanism of each PKC isoform in relation to cellular functions.

In this study, we observed downregulation of ACOT7 upon treatment with genotoxic stresses such as ionizing radiation (IR) and doxorubicin (Doxo). We found that ACOT7 depletion induced cytostasis through the PKC*ζ*–p53–p21 signaling pathway. Furthermore, we demonstrated the synergistic effect of ACOT7 depletion and treatment with either IR or Doxo on cancer cell proliferation, suggesting ACOT7 might be a novel target for anti-cancer therapy.

## Results

### ACOT7 is downregulated in cells exposed to IR or Doxo

**Figure 1:**
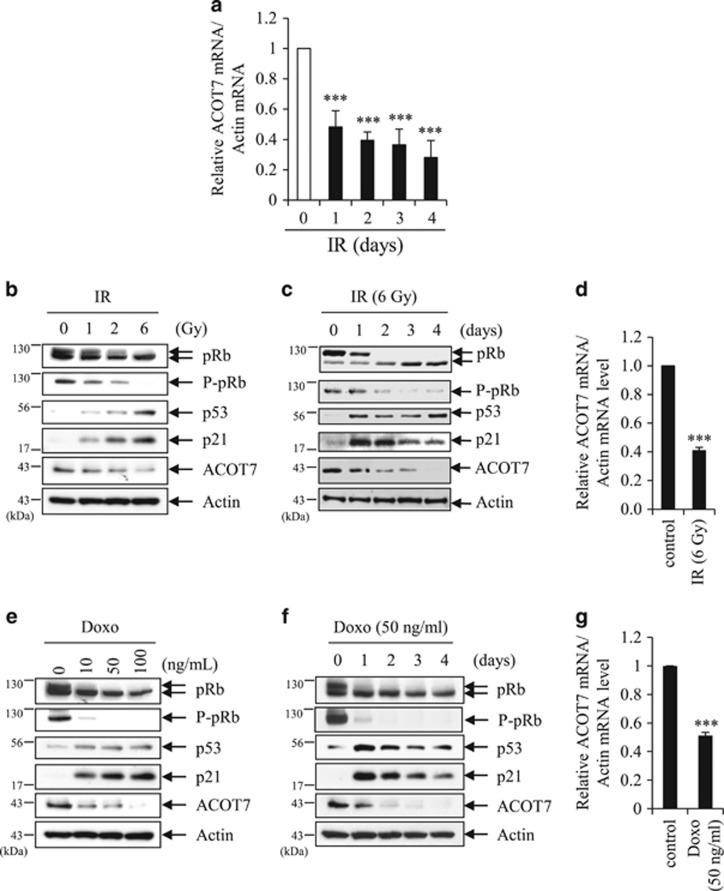
ACOT7 is downregulated in IR-exposed and Doxo-treated cells. (**a**) Reduction of ACOT7 in IR-exposed MCF7 cells. Cells were exposed to 6 Gy of IR and harvested at the indicated time, followed by microarray. Levels of ACOT7 mRNA in IR-exposed cells were compared with that of the unexposed control group. (**b** and **c**) MCF7 cells were exposed to IR at doses of 1, 2, and 6 Gy and harvested 2 days after irradiation (**b**) or exposed to 6 Gy of IR and harvested on the indicated days after irradiation (**c**), followed by immunoblotting. Actin was used as a loading control. (**d**) MCF7 cells were irradiated with 6 Gy of IR, incubated for 2 days, and then harvested for qRT-PCR analyses. (**e** and **f**) MCF7 cells were treated with 10, 50, and 100 ng/ml of Doxo and harvested 2 days after treatment (**e**) or treated with 50 ng/ml of Doxo and harvested on the indicated days after treatment (**f**), followed by immunoblot analysis. Actin was used as a loading control. (**g**) MCF7 cells were treated with 50 ng/ml of Doxo, incubated for 2 days, and then harvested for qRT-PCR analyses. The values represent the mean±S.D. of three independent experiments. *** Indicates the statistical significance of *P*<0.001 by Student’s *t*-test

To examine changes in the gene expression profile of irradiated cancer cells, we conducted gene expression analysis of an IR-exposed MCF7 human breast carcinoma.^35^ Gene expression analysis showed that ACOT7 expression markedly decreased in IR-exposed MCF7 cells (Figure 1a). To verify downregulation of ACOT7 upon IR exposure, MCF7 cells were exposed to various doses of IR (1, 2, and 6 Gy), and protein levels of ACOT7 were measured using western blot analysis. Levels of ACOT7 protein decreased in IR-exposed MCF7 cells in dose- and time-dependent manners (Figures 1b and c). From the qRT-PCR results, reduction of ACOT7 mRNA expression was confirmed in IR-exposed cells (Figure 1d). To determine whether or not ACOT7 expression was altered after treatment with the anti-cancer drug Doxo, we measured ACOT7 mRNA and protein levels in Doxo-treated MCF7 cells. Levels of ACOT7 protein were also reduced in Doxo-treated cells in dose- and time-dependent manners (Figures 1e and f). ACOT7 mRNA expression also evidently decreased in Doxo-treated MCF7 cells (Figure 1g). When we assessed apoptotic cells with Annexin V positivity, the applied doses of IR (1, 2, and 6 Gy) and of low concentration Doxo (10, 50, and 100 ng/ml) did not significantly induce apoptosis compared to that in 5 *μ*g/ml Doxo-treated cells as a positive control (Supplementary Figure S1). To determine whether other ACOT family members could be induced by IR or Doxo treatment, we examined the changes in expression levels of ACOT1, 4, 8, 9, 11, 12, and 13 which are expressed in human, as is ACOT7.^2^ ACOT4 and 12 were overexpressed and ACOT8 was underexpressed in IR- and Doxo-treated MCF7 cells (Supplementary Figure S2a).

### ACOT7 depletion induces cytostasis via p53–p21 signaling pathway

**Figure 2:**
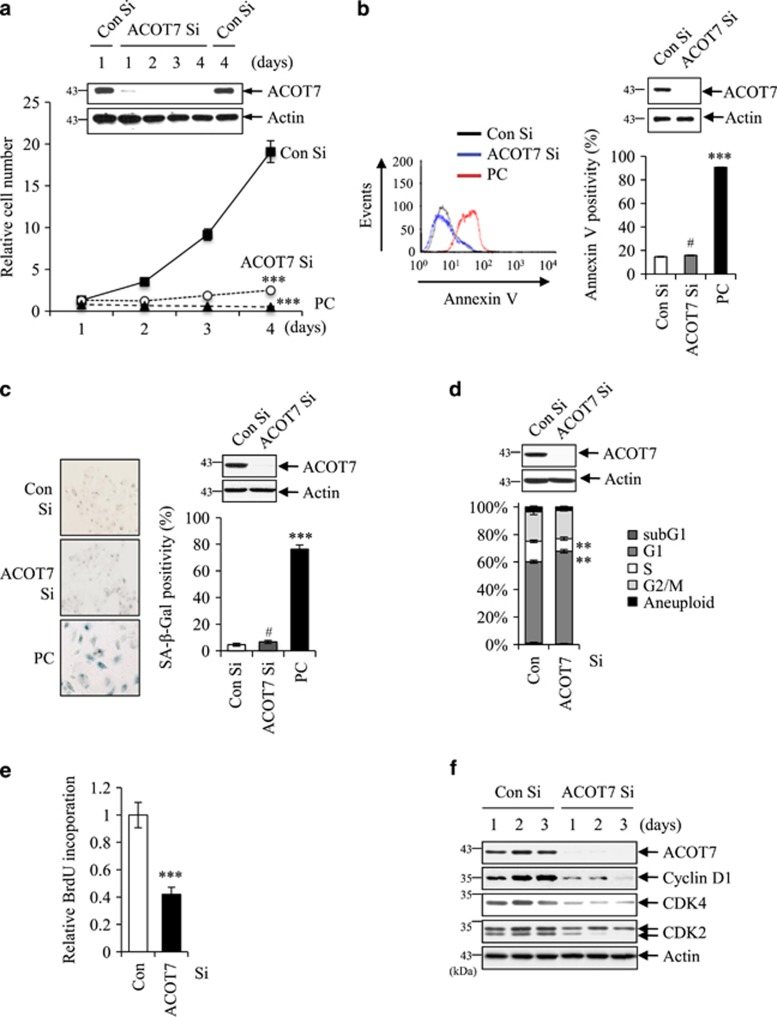
Depletion of ACOT7 induces cell cycle arrest. (**a**) Relative numbers of viable cells were determined on the indicated days after transfection of 100 nM ACOT7 Si into MCF7 cells. Viable cell numbers on 1 day after transfection was considered as 1. Cells treated with Doxo (5 *μ*g/ml) were used as a positive control (PC). Total protein was extracted from cells the indicated days after siRNA transfection and was subjected to WB analysis. (**b**) Percentages of positive cells for Annexin V staining were determined in MCF7 cells transfected with Con Si and ACOT7 Si using fluorescence-activated cell sorting (FACS) 1 day after transfection. Cells treated with Doxo (5 *μ*g/ml) were used as a PC for apoptosis. (**c**) Positivity for SA-*β*-Gal staining was evaluated at 4 days after transfection of either Con Si or ACOT7 Si into MCF7 cells. Cells exposed to IR (6 Gy) were used as a PC for SA-*β*-Gal staining. (**d**) Cells were harvested 3 days after transfection with Con Si and ACOT7 Si, and FACS was used to analyze cell cycle distribution. (**e**) DNA synthesis rates of MCF7 cells with ACOT7 depletion were measured based on BrdU incorporation into cells. (**f**) Whole-cell lysates were prepared from cells transfected with Con Si and ACOT7 Si on the indicated days after transfection. Immunoblotting was performed. Actin served as a loading control. The values represent the mean±S.D. of three independent experiments. ***, **, and ^#^ indicate the statistical significance of *P*<0.001, *P*<0.01, and *P*>0.05 by Student’s *t*-test, respectively, comparing to cells transfected with either Con Si

Next, we assessed the effect of ACOT1, 4, 7, 8, 9, 11, 12, and 13 knockdown and their effects on relative cell numbers in MCF7 cells (Supplementary Figures S2b and c). As shown in Supplementary Figure S2c, only ACOT7 depletion produced a marked decrease of relative cell numbers, although ACOT9 showed a slight decrease of relative cell numbers. Thus, we transfected siRNA against ACOT7 (ACOT7 Si) into MCF7 cells and further observed the effect of ACOT7 depletion on cell proliferation and survival. MCF7 cells treated with ACOT7 Si decreased in number compared with Control siRNA (Con Si) treatment up to 4 days (Figure 2a). Reduction of cell number could be primarily attributed to induction of apoptosis or cellular senescence or cytostasis. MCF7 cells transfected with ACOT7 Si did not show elevation of Annexin V positivity (marker of apoptosis) (Figure 2b). High concentration of Doxo (1 *μ*g/ml) was used as a positive control (PC) of apoptosis (Figures 2a and b). Next, we examined whether or not ACOT7 depletion could trigger cellular senescence. Positivity of SA-*β*-galactosidase (SA-*β*-Gal), a marker of cellular senescence, was not affected by ACOT7 depletion, in contrast to the PC group showing induction of cellular senescence upon 6 Gy of IR exposure (Figure 2c). ACOT7-depleted MCF7 cells exhibited a higher proportion of cells in G1 phase as well as less cells in S phase (Figure 2d). BrdU incorporation was reduced to 40% of that of the control (Con) group (Figure 2e). Consistent with these results, proteins involved in cell cycle regulation such as cyclin D1, cyclin-dependent kinase 2 (CDK2), and CDK4 were also downregulated in ACOT7-depleted cells (Figure 2f). These results demonstrate that ACOT7-depleted cells underwent cytostasis rather than apoptosis or cellular senescence. However, when ACOT7 was overexpressed in MCF7 cells, cell morphology (Supplementary Figure S3a), cell number (Supplementary Figure S3b), and levels of phosphorylated pRb (P-pRb), p53, and p21 (Supplementary Figure S3c) were not affected.

**Figure 3:**
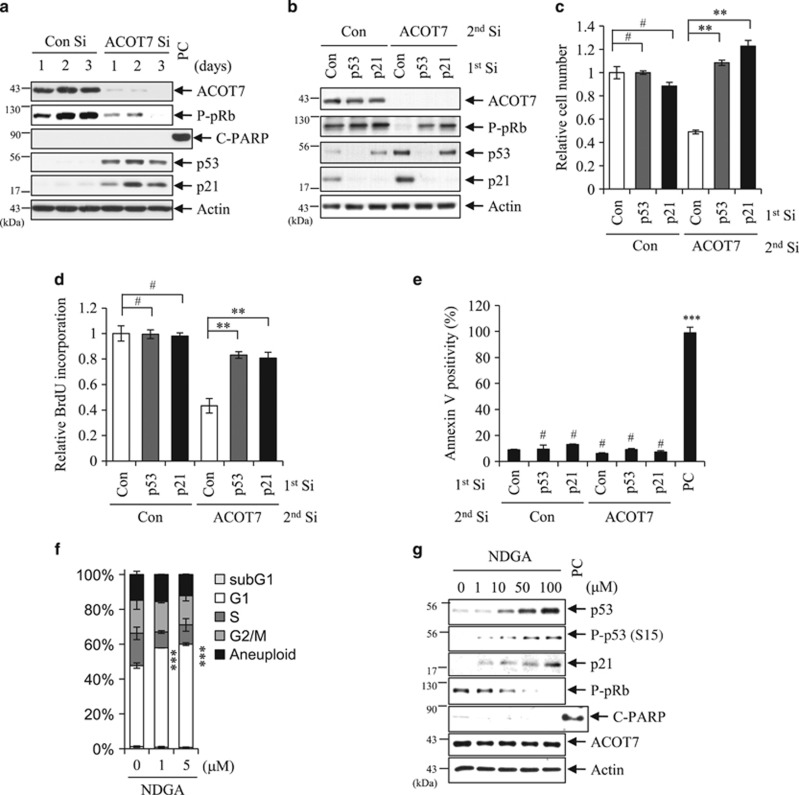
Cell cycle arrest mediated by ACOT7 depletion requires p53 and p21. (**a**) MCF7 cells were transfected with Con Si and ACOT7 Si. Cells were harvested on the indicated days after transfection. Actin was used as a loading control. (**b**) MCF7 cells were transfected with Con Si, p53 Si, or p21 Si. On the next day, half of each group was transfected with Con Si while the other half was transfected with ACOT7 Si. Two days after the second transfection, whole-cell extracts were prepared from cells, and immunoblotting was performed. Actin served as a loading control. (**c**) Relative numbers of viable cells were determined 4 days after transfection with the indicated siRNAs. (**d**) BrdU incorporation rates were determined in cells transfected with the indicated siRNAs. (**e**) FACS of siRNA-transfected cells was used to assess percentages of Annexin V-positive cells. (**f** and **g**) MCF7 cells were harvested 3 days after NDGA (acyl-CoA thioesterase inhibitor) treatment. FACS was used to analyze the cell cycle distribution (**f**), and immunoblotting was performed (**g**). Cells treated with 5 *μ*g/ml of Doxo served as a PC. The values represent the mean±S.D. of three independent experiments. ***, **, and ^#^ indicate the statistical significance of *P*<0.001, *P*<0.01, and *P*> 0.05 by Student’s *t*-test, respectively

Depletion of ACOT7 in MCF7 cells was accompanied by p53 and p21 accumulation in close correlation with reduction of P-pRb (Figure 3a). Poly (ADP-ribose) polymerase (PARP) cleavage as a marker of apoptosis was not detected, consistent with the results of Figure 2b. To determine whether or not cytostasis in ACOT7-depleted cells was directly correlated with either p53 accumulation or p21 induction, we knocked down either p53 or p21 prior to ACOT7 depletion using specific siRNAs (Figure 3b). Induction of p21 was completely dependent on p53 activation in ACOT7-depleted cells (Figure 3b). While depletion of ACOT7 alone markedly decreased cell number, depletion of ACOT7 in combination with either p53 Si or p21 Si rescued cell number up to that of the control group (Figure 3c). Depletion of either p53 or p21 alone did not significantly affect relative cell number compared to the control group (Figure 3c). BrdU incorporation was closely correlated with relative cell numbers in p53- and p21-depleted cells in the presence or absence of ACOT7 (Figure 3d). Under such conditions, Annexin V positivity was not affected (Figure 3e). We also observed a dramatic decrease in cell numbers in ACOT7-depleted A549 human lung carcinoma cells due to cycle arrest compared to Con Si-treated cells (Supplementary Figures S4a and b). ACOT7-depleted A549 cells exhibited hypo-phosphorylation of pRb and activation of the p53/p21 signaling pathway (Supplementary Figure S4c).

ACOT7 is a cytosolic acyl coenzyme A thioester hydrolase that primarily hydrolyzes the CoA thioester of palmitoyl-CoA. To determine whether or not ACOT7 depletion-mediated cytostasis is due to acyl-CoA thioesterase activity *per se*, we treated MCF7 cells with nordihydroguaiaretic acid (NDGA), an inhibitor of Acyl-CoA thioesterase.^36^ NDGA treatment resulted in cell cycle arrest in G1 phase (Figure 3f). NDGA treatment induced hypophosphorylation of pRb as well as p53 and p21 accumulation but no detectable PARP cleavage in dose-dependent manner (Figure 3g). These results indicate that loss of Acyl-CoA thioesterase activity could trigger the p53–p21 signaling pathway, resulting in cell cycle arrest in cancer cells.

**Figure 4:**
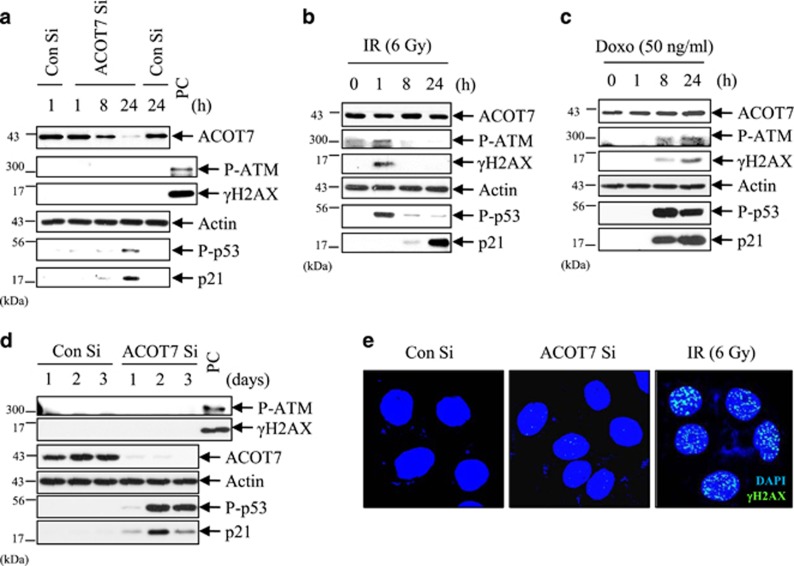
The DNA damage response is not involved in ACOT7 depletion-mediated cytostasis. (**a**–**d**) After transfection of ACOT7 Si (**a** and **d**), or after exposure to 6 Gy IR (**b**), or after treatment with 50 ng/ml Doxo (**c**), MCF7 cells were harvested at the indicated time intervals. Western blotting analysis was performed with anti-phospho ATM and anti-*γ*H2AX. (**e**) Immunocytochemical staining of *γ*H2AX (green) was conducted at 1 day after transfection of ACOT7 Si. DNA was counterstained with DAPI (blue). Cells exposed to 6 Gy of IR were used as the positive control (PC)

We further examined whether the DNA damage response is triggered during ACOT7 depletion-induced cytostasis. We performed western blot analyses with anti-phospho ATM and anti-gamma-H2AX antibodies to detect the DNA damage response in ACOT7-depleted MCF7 cells. ACOT7 depletion did not change the phosphorylation status of ATM or *γ*-H2AX at short (1, 8, and 24 h) and longer (1, 2, and 3 d) period time intervals (Figures 4a and d). In contrast, phosphorylations of ATM and *γ*-H2AX were detected in both 6 Gy of IR-exposed and 50 ng/ml Doxo-treated positive control groups (Figures 4b and c). We also examined *γ*-H2AX foci in nucleus, an indicator of DNA damage, with confocal microscope. In contrast to IR-exposed cells, ACOT7 Si-transfected cells did not exhibit gamma-H2AX foci in the nucleus (Figure 4e). These results indicate that ACOT7 depletion induces cytostasis in the absence of a DNA damage response.

### PKC*ζ* is involved in ACOT7 depletion-mediated cell cycle arrest

**Figure 5:**
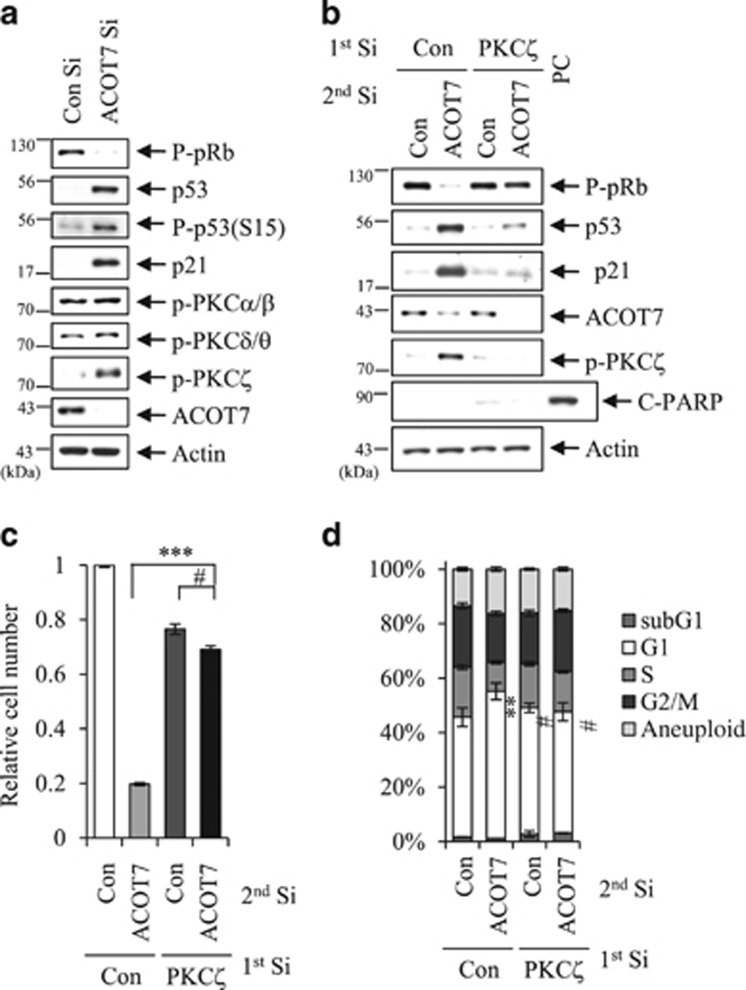
Activation of PKC*ζ* is involved in cell cycle arrest induced by ACOT7 depletion. (**a**) Cells were harvested 2 days after transfection with PKC*ζ* Si, after which immunoblotting was performed. Actin served as a loading control. (**b**–**d**) MCF7 cells were transfected with Con Si or PKC*ζ* Si. On the next day, cells were transfected with Con Si or ACOT7 Si. Transfected cells were harvested for immunoblotting (**b**), the relative numbers of viable cells (**c**), and FACS analysis for cell cycle distribution (**d**) in cells transfected with the indicated siRNAs. Two days of transfection, immunoblotting and FACS analysis were conducted. Actin served as a loading control. Four days after transfection, viable cells were counted and compared with that of the control group, which is 1. Cells treated with 5 *μ*g/ml of Doxo served as a PC. The values represent the mean±S.D. of three independent experiments. *** and ^#^ indicate statistical significance of *P*<0.001 and *P*>0.05 by Student’s *t*-test, respectively

Next, we identified which upstream molecule induced p53 activation under ACOT7-depleted conditions. ACOT7 produces arachidonic acid (AA) and CoA-SH from arachidonoyl-CoA.^37^ and AA production might be associated with PKC activity.^20, 38, 39^ To determine whether or not PKC activity is involved in activation of the p53–p21 signaling pathway induced by ACOT7 depletion, we analyzed the phosphorylation status of several PKC subtypes. While phosphorylation of PKC*α/β* and *δ/θ* was not altered by ACOT7 depletion, PKC*ζ* phosphorylation was evidently induced in ACOT7-depleted cells (Figure 5a). To rule out the possibility of an off-target effect of ACOT7 Si, we transfected another ACOT7 Si sequence (ACOT7 #2). We confirmed a lack of off-target effects of ACOT7 Si in pRb hypo-phosphorylation, p53–p21 accumulation, and PKC*ζ* activation (Supplementary Figure S4d). We also observed PCK*ζ* phosphorylation as well as pRb hypo-phosphorylation and activation of the p53/p21 signaling pathway in ACOT7-depleted A549 cells (Supplementary Figure S4c). To examine direct involvement of PKC*ζ* in activation of the p53–p21 signaling pathway induced by ACOT7 depletion, we co-transfected PKC*ζ* Si and ACOT7 Si into MCF7 cells. We failed to detect hypo-phosphorylation of pRb and activation of the p53–p21 signaling pathway in ACOT7 and PKC*ζ* double knock downed cells (Figure 5b). While cells transfected with ACOT7 Si showed decreased cell numbers and cell cycle arrest in G1 phase, cells co-transfected with ACOT7 Si and PKC*ζ* Si recovered relative cell numbers and were released from cell cycle arrest in G1 phase compared with either control cells or PKC*ζ*-alone transfected cells (Figures 5c and d).

Next, we investigated whether, in addition to ACOT7, other isoforms of ACOT family members including ACOT1, 4, 8, 9, 11, 12, and 13 are also able to activate the PCK*ζ*–p53–p21 signaling pathway and induce cell cycle arrest (Supplementary Figure S2d). In contrast to the effect of ACOT7 depletion, depletion of other ACOT family members did not affect phosphorylation of PKC*ζ* nor induced activation of the p53–p21 signaling pathway (Supplementary Figure S2d). Among the tested ACOT family members, hypophosphorylation of pRb was the most evident in ACOT7-depleted cells. ACOT9 depletion showed p21 accumulation and ACO11 depletion induced PKC*α/β* phosphorylation. These results indicate that ACOT7 depletion induced cell cycle arrest specifically through activation of the PKC*ζ*–p53–p21 signaling pathway; other members of the ACOT family did not.

### ACOT7 plays role as anti-tumor therapeutic target

**Figure 6:**
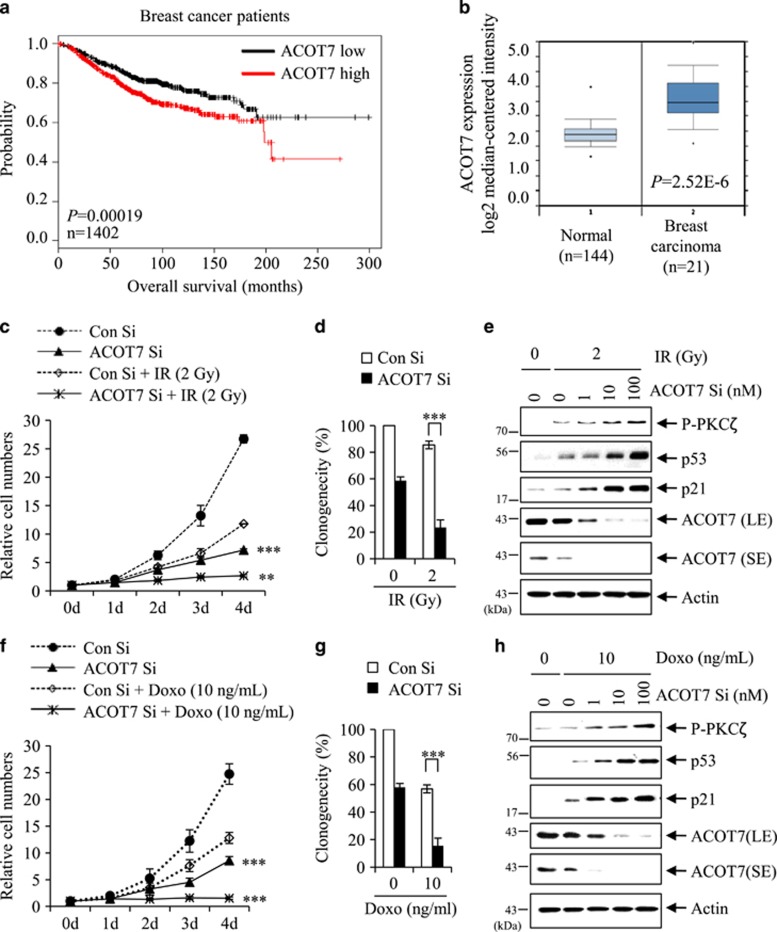
ACOT7 depletion sensitizes breast cancer cells to irradiation and anti-cancer drug. (**a**) Kaplan–Meier curves of overall survival times of patients with breast cancer. Data were obtained from http://kmplot.com/analysis/. Statistical significance was determined using the log-rank test. (**b**) Box plots comparing ACOT7 expression (as log2 median-centered ratios) in normal breast and carcinoma breast tissues. Dots indicate extreme data values. Data were obtained from http://oncomine.org/. (**c** and **d**) MCF7 cells were transfected with Con Si or 10 nM ACOT Si prior to 2 Gy of IR exposure. Relative cell numbers were determined on the indicated days (**c**). Colony-forming assay was performed after 7 days (**d**). (**e**) Immunoblot analysis were performed on MCF7 cells transfected with ACOT7 Si indicated concentrations and exposed to 2 Gy of IR. Actin was used as a loading control. (**f** and **g**) MCF7 cells were transfected with Con Si or 10 nM ACOT Si prior to treatment with 10 ng/ml of Doxo. Relative cell number was determined on the indicated days (**f**). Colony-forming assay was performed after 7 days (**g**). (**h**) Immunoblot analysis was performed in MCF7 cells transfected with ACOT7 Si and then treated with 10 ng/ml of Doxo for 2 days. Actin was used as a loading control. SE and LE indicates short and long exposures, respectively. The value represents the mean±S.D. from three independent experiments. *** and ** indicate statistical significance of *P*<0.001 and *P*<0.01 by Student’s *t*-test, respectively

To explore the biological significance of the critical role of ACOT7 in cell cycle progression, we evaluated the prognostic value of ACOT7 expression using microarray data from patients with breast cancer (http://kmplot.com/analysis/). Patients with low ACOT7 levels showed longer overall survival periods than breast cancer patients with high ACOT7 levels (Figure 6a). We also analyzed gene expression data from the Cancer Genome Atlas and observed upregulated ACOT7 expression in carcinoma breast tissues compared with normal breast tissues (Figure 6b). To evaluate the anti-tumor effect of ACOT7 depletion in combination with IR in breast carcinoma cells, ACOT7 was depleted in MCF7 cells using siRNA prior to exposure to 2 Gy of IR. As shown, effects of ACOT7 Si in combination with 2 Gy of IR on cell growth and clonogenicity were more effective than 2 Gy of IR (PC) alone (Figures 6c and d). Downregulation of ACOT7 by transfection of ACOT7 Si in combination with 2 Gy of IR exposure further increased accumulation of p53 and p21 via PKC*ζ* activation comparing to 2 Gy of IR alone (Figure 6e). We also examined the anti-tumor effect of ACOT7 depletion in combination with Doxo (10 ng/ml) and found that combined ACOT7 Si and Doxo treatment increased anti-cancer drug sensitivity as well as accumulation of p53 and p21 via PKC*ζ* activation in MCF7 cells (Figures 6f–h).

**Figure 7:**
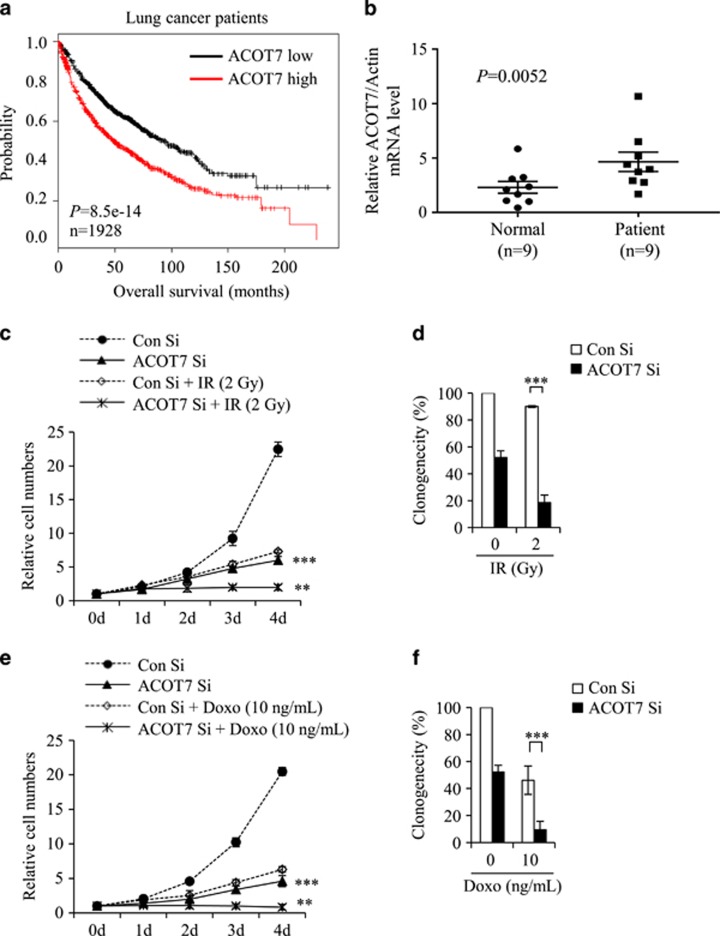
Effect of ACOT7 depletion in irradiated and anti-cancer drug-treated lung cancer cells and patients. (**a**) Kaplan–Meier curves of overall survival times of patients with lung cancer. Data were obtained from http://kmplot.com/analysis/. Statistical significance was determined using the log-rank test. (**b**) Quantitative RT-PCR analysis of ACOT7 mRNA in patient tissues. RNA was purified from lung cancer tissues and corresponding normal counterparts from nine patients and subjected to qRT-PCR using ACOT7-specific primers. Expression levels were normalized to actin mRNA. Error bars represent ±S.E.M. (**c** and **d**) A549 cells were transfected with Con Si or 10 nM ACOT Si prior to exposure to 2 Gy of IR. Relative cell numbers were determined on the indicated days (**c**). Colony-forming assay was performed after 7 days (**d**). (**e** and **f**) A549 cells were transfected with Con Si or 10 nM ACOT Si prior to treatment with 10 ng/ml of Doxo. Relative cell numbers were determined on the indicated days (**e**). Colony-forming assay was performed after 7 days (**f**). The value represents the mean±S.D. from three independent experiments. *** and ** indicate statistical significance of *P*<0.001 and *P*<0.01 by Student’s ***t***-test, respectively

**Figure 8:**
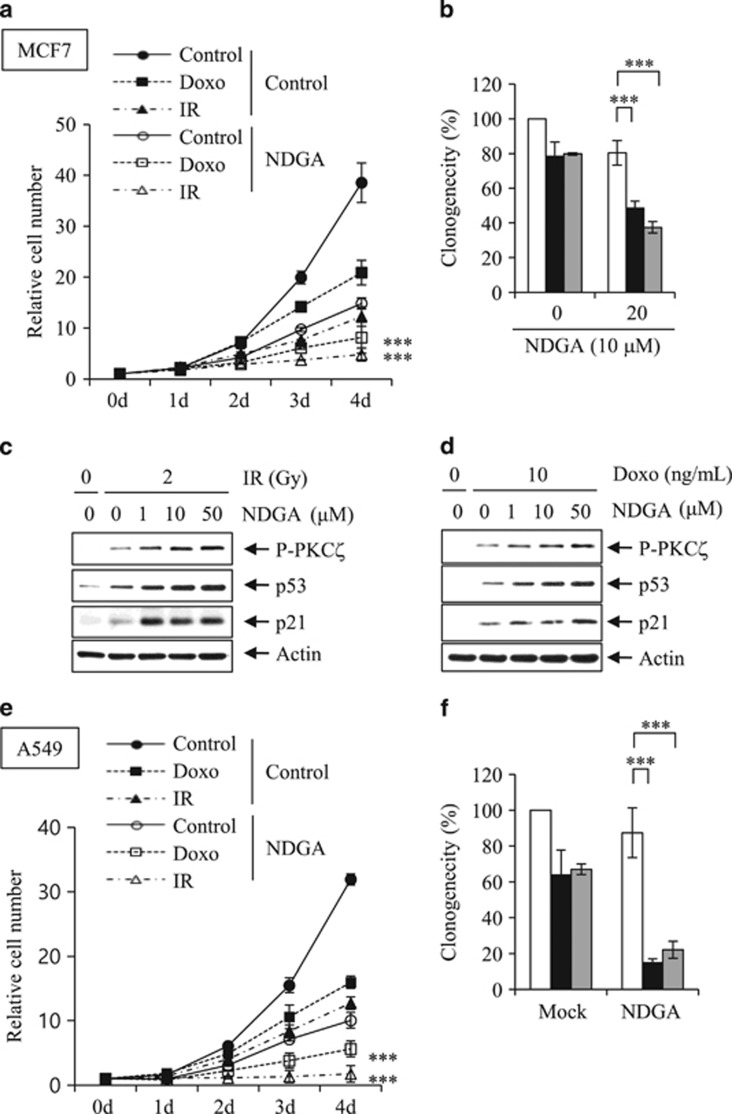
Synergistic anti-tumoral effect of NDGA treatment in combination with IR or Doxo. MCF7 cells were treated with NDGA prior to exposure to 2 Gy of IR or to treatment with 10 ng/ml of Doxo. (**a**–**d**) Relative cell numbers were determined at the indicated time intervals (**a**), colony-forming assay was performed at 7 days (**b**), and western blot analyses were performed in MCF7 cells 2 days (**c** and **d**) after IR or Doxo treatment. (**e** and **f**). A549 cells were treated with NDGA prior to exposure to 2 Gy of IR or treatment with 10 ng/ml of Doxo. Relative cell numbers were determined at the indicated time intervals (**e**), and colony-forming assay was performed at 7 days (**f**) after IR or Doxo treatment. NDGA concentration was 10 *μ*M (**a**, **b**, **e**, and **f**) and actin was used as the loading control (**c** and **d**). *** and ** indicate statistical significance of *P*<0.001 and *P*<0.01, respectively, as determined by Student’s *t*-test

Low ACOT7 levels also prolonged overall survival periods in lung cancer patients based on analysis of the ACOT7 prognostic value (http://kmplot.com/analysis/) (Figure 7a). Furthermore, we assessed mRNA levels of ACOT7 in lung cancer patient tissues. We collected cancerous and corresponding normal tissue samples from nine lung cancer patients diagnosed with adenocarcinoma, bronchioalveolar carcinoma, or squamous cell carcinoma. Relative mRNA expression levels were analyzed using qRT-PCR (Figure 7b). Cancer tissues exhibited higher ACOT7 mRNA levels than normal tissues (*P*=0.0052; data are presented as mean±S.E.M.). To assess the anti-tumor effect of ACOT7 depletion in combination with either IR or Doxo, we measured relative cell numbers and clonogenicity in ACOT7-depleted A549 human lung carcinoma cells. As shown, ACOT7 depletion induced tumor sensitivity either to IR (Figures 7c and d) or Doxo (Figures 7e and f). Furthermore, we evaluated the anti-tumoral effect of NDGA treatment in combination with IR or Doxo in MCF7 breast carcinoma cells. As shown in Figure 8, NDGA treatment enhanced the effect of IR or Doxo on cell growth and clonogenicity comparing with IR- or Doxo-alone-treated cells (Figures 8a and b). In addition, combination treatments of NDGA with either IR or Doxo were augmented PKC*ζ*–p53–p21 signaling pathway (Figures 8c and d). We also examined the anti-tumoral effect of NDGA treatment in combination with IR or Doxo in A549 lung carcinoma cells, and found the enhanced anti-tumoral effects comparing to IR- or Doxo-alone-treated cells (Figures 8e and f). Altogether, these results indicate that a decreased ACOT7 activity may be involved, at least in part, in prevention of human breast and lung cancer development via regulation of cell cycle progression.

## Discussion

Radiotherapy and chemotherapy are the most effective therapeutic regimes along with surgery for cancer treatment.^40^ Many studies have focused on increasing tumor sensitivity to IR and chemotherapeutic drugs. We have also tried to identify genes that are specifically involved in cellular responses to IR and chemotherapeutic drugs.^35, 41, 42^ Here we observed that ACOT7 was responsive to DNA damaging stimuli such as IR and Doxo in breast and lung carcinoma cells (Figure 1). When we transfected ACOT7 Si alone, cancer cell proliferation was markedly inhibited via activation of the PKC*ζ*–p53–p21 signaling pathway (Figure 2). In addition, treatment with ACOT7 Si in combination with either IR or Doxo further sensitized cancer cells through augmentation of the PKC*ζ*–p53–p21 signaling pathway (Figures 6 and 7). ACOT7 depletion as well as the acyl-CoA thioesterase inhibitor NDGA induced cell cycle arrest through induction of p53–p21 accumulation (Figure 3), indicating that acyl-CoA thioesterase activity *per se* might be involved in cell cycle regulation.

PKC is a family of serine/threonine kinases with at least 10 isoforms. The PKC family has been identified as a key player that orchestrates downstream signaling pathways regulating apoptosis and cell survival.^23, 43, 44, 45^ However, PKC has a striking feature that individual isoforms can exert either similar or opposite effects in cellular processes. Moreover, although PKCs are the key enzymes involving in p53 phosphorylation, PKC isoforms differentially regulate p53 activity.^34^ In this study, PKC*ζ* was found to be specifically involved in ACOT7-mediated p53 activation, p21 accumulation, and cell cycle arrest, whereas PKC*α/β* and *δ/θ* were not (Figure 4). Our results support that PKC isoforms differentially regulate p53 activity and have their own specific features in cellular processes.

ACOTs regulate lipid metabolism by modulating cellular levels of activated fatty acids (acyl-CoAs), free fatty acids, and CoA.^1^ The ACOT family consists of 13 mammalian genes and is divided into two subfamilies base on enzyme weight.^2, 3^ Type I ACOTs comprise the smaller group, which contains only four genes: ACOT1, ACOT2, ACOT4, and ACOT6 in human. ACOT3 and ACOT5 are found in both mouse and rat.^3^ ACOT1 and ACOT2 are the most closely related, having 98% amino-acid sequence identity. ACOT6 mRNA is not detected in human tissue, and only a truncated version of the protein could be expressed by using of recombinant clone.^2^ Type II ACOTs are strikingly less related than type I proteins. ACOT10 has only been identified in mice. Within the ACOT enzyme family, in particular, ACOT7 has been identified as having an important function in inflammatory reactions through production of AA.^2^ It was proposed that ACOT7-mediated AA production may provide a complementary source of AA to the well-characterized phospholipase A_2_ (PLA_2_) pathway.^9, 46^ The high activity of ACOT7 toward arachidonoyl-CoA may also suggest a role for thioesterase in signal transduction, possibly via PKC activation.^1^ To date, however, relatively few studies have examined the pathophysiological function of ACOT7, and no study has reported the association between ACOT7 and PKC*ζ* in cellular processes. In order to verify that activation of the PKC*ζ*–p53–p21 signaling pathway by depletion of ACOT7 was not due to altered concentrations of free fatty acids and CoA, we depleted other members of ACOT family which are expressed in human (ACOT 1, 4, 8, 9, 11, 12, and 13) to determine their effect on cell cycle and the PKC*ζ*–p53–p21 signaling pathway (Supplementary Figure S5). We did not include ACOT2 because it has 98% amino-acid sequence identity with ACOT1. As shown in Supplementary Figure S5, depletion of other isoforms of ACOT did not affect the PCK*ζ*–p53–p21 signaling pathway. The results reveal that ACOT7 among the tested ACOT family members has a role in cell cycle progression through inhibition of PKC*ζ*–p53–p21 signaling pathway.

Among ACOT family members, ACOT1 (cytosolic), ACOT2 (mitochondrial), and ACOT7 (cytosolic) have been well characterized for their enzymatic properties.^4^ These ACOTs share a common feature in that their expression is markedly induced by ligands for peroxisome proliferator-activated receptor *α* (PPAR*α*), a nuclear receptor that regulates lipid metabolism-related genes such as the fibrate class of hypolipidemic drugs.^4^ Moreover, ACOT1 and ACOT2 have been implicated in fatty acid catabolism, as emphasized by their upregulation in the liver and heart of fasted or high-fat-diet fed animals.^47, 48, 49, 50^ However, ACOT7 has two orders of magnitude higher enzyme activity than ACOT1 and ACOT2, and its expression is prominent in neurons of the central and peripheral nervous systems.^15, 18^ ACOT7 may play a role in cellular events other than energy metabolism, as overexpression of ACOT7 modified production of prostaglandins in a macrophage cell line.^9^ Other members of the ACOT family that possess substrate specificity for short, medium, and long acyl-CoA were not involved in cell cycle progression through the regulation of the PKC*ζ*–p53–p21 signaling pathway (Supplementary Figure S2d), thus implying that the role of ACOT7 might be driven by alteration of the free AA level rather than the cellular CoA level. However, secondary changes induced by ACOT7 depletion cannot be ruled out as a cause for activation of the PKC*ζ*–p53–p21 signaling pathway. Further studies examining the molecular mechanism of ACOT7 will be required to fully determine how ACOT7 is involved in PKC*ζ* activation and how PKC*ζ* induces p53 accumulation.

In the present study, for the first time, we demonstrated that ACOT7 plays a critical role in cell cycle progression. ACOT7 depletion induces cell cycle arrest through regulation of the PKC*ζ*–p53–p21 signaling pathway. We demonstrated that ACOT7 might be a valuable target for radiotherapy and chemotherapy *in vitro*. In addition, the antitumor effect of ACOT7 depletion is supported by analysis of the prognostic value and its higher expression level in lung cancer patient tissues than normal tissues. Our data reveal a non-canonical function of ACOT7 as a critical cell cycle regulator and a novel aspect of acyl-CoA thioesterase as a novel target for anticancer therapy.

## Materials and Methods

### Culture of cells

MCF7 and A549 cells were obtained from the American Type Culture Collection (ATCC) (Manassas, VA, USA) and grown in Dulbecco’s modified Eagle’s medium (PAA Laboratories GmbH, Pasching, Austria) and RPMI 1640 (WelGENE, Daegu, Korea), respectively, containing 10% FBS (Lonza Group Ltd., Basel, Switzerland) and 1% penicillin and streptomycin solution (WelGENE) at 37 °C under 5% CO_2_.

### Irradiation

Cells were exposed to a ^137^Cs *γ*-ray source (Atomic Energy of Canada Ltd., Chalk River, Ontario, Canada) at a dose rate of 3.2 Gy/min.

### Determination of relative number of viable cells

Cells were stained with trypan blue, and those excluded from staining (viable cells) were counted under a microscope using a hemocytometer. The number of viable cells prior to treatments was regarded as 1, and the number of viable cells of treatment groups were displayed as a relative number compared to the control group.

### RNA interference

Sequences of siRNAs for p21, p53, and the non-specific siRNA were described previously.^51^ The siRNAs for ACOT7 (5′-AGACCGAGGACGAGAAGAAdTdT-3′), ACOT7#2 (5′-GUGCAGGUCAACGUGAUGUdTdT-3′), ACOT1 (5′-UUAAAGAAGCUGUGAACUAdTdT-3′), ACOT4 (5′-GAGAGAUUCAAGAUCAGAUdTdT-3′), ACOT8 (5′-CGAAGCUGCCAGUACUGUAdTdT-3′), ACOT9 (5′-GAUAAGUUGCGGGAGAUAGdTdT-3′), ACOT11 (5′-GCACACCAUUAGUGUUGGAdTdT-3′), ACOT12 (5′-GGUGGCUGGUCAAAAUCCAdTdT-3′), ACOT13 (5′-GAGGAAAGTCAGTGAGCAAdTdT-3′) and PKC*ζ* (5′-GACAUGAACACAGAGGACUACCdTdT-3′) were obtained from Bioneer (Daejun, Korea). MCF7 cells were transfected with 100 nM siRNA using RNAi-MAX (Invitrogen, Carlsbad, CA, USA) according to the manufacturer’s instructions.

### Human tissue collection and qRT-PCR analysis

This study involving human tissues was approved by the Asan Medical Center Institutional Review Board (IRB Approval No. K‐1007‐002‐067). Snap‐frozen tissues from lung tissues were obtained from patients with lung cancer that had been diagnosed as lung adenocarcinoma, squamous cell carcinoma, or bronchioloalveolar carcinoma. Total RNA was isolated from tissues and cells using TRIzol reagent (Invitrogen). Semi-quantitative (sq) RT-PCR was performed as described previously^47^ using gene-specific primers:

ACOT7, 5′-CTGCACCCTGCACGGCTTTG-3′ (sense) and 5′-CGGAAGCTGTGACGATGTTG-3′ (antisense); ACOT1, 5′-CTGCCCTAGTGGGCGTTTAG-3′ (sense), and 5′-GAACCCAAATAATCCGGCCA-3′ (antisense); ACOT4, 5′-AGGAGGGTACAAGAACCCCA-3′ (sense) and 5′- GAGGCTCGATGTAATGCCCA-3′ (antisense); ACOT8, 5′-GCCGCCTATATCTCCGACTAT-3′ (sense) and 5′-GCTCTCGCATTCATAGAGCAT-3′ (antisense); ACOT9, 5′-AAGTTCAGTGGCCATGTTAGC-3′ (sense) and 5′-AATGCCGGCCCTTTATTTTCA-3′ (antisense); ACOT11, 5′-GAGATGGTGGTGCATGTGGA-3′ (sense) and 5′-CGTCTGTACTCTGGCGTCTC-3′ (antisense); ACOT12, 5′-GCTGGAAATCTTGGTGGCTG-3′ (sense) and 5′-GCCCCAAAGCTTGACAGATG-3′ (antisense); ACOT13, 5′-CTCTTCGCCCTTTGTGTCCT-3′ (sense) and 5′-GAGTAATCTTTCCCAAAACTCTCTC-3′ (antisense) and actin, 5′-CAAGAGATGGCCACGGCT-3′ (sense) and 5′-TCCTTCTGCATCCTGTCGGC-3′ (antisense).

### Immunoblot analyses

Immunoblotting was performed as described previously.^50^ Briefly, whole-cell extracts or immunopurified proteins were separated on sodium dodecyl sulfate-polyacrylamide gels (6–12%) and transferred to a HyBond ECL nitrocellulose membrane (GE Healthcare, Little Chalfont, UK). The following antibodies were used as primary antibodies: anti-ACOT7 (Abcam, Cambridge, UK), anti-pRb (Cell Signaling, Danvers, MA, USA), anti-phospho-pRb (Cell Signaling), anti-cleaved PARP (Cell Signaling), anti-p53 (Do-7; Leica, Milton Keynes, UK), anti-p21 (Santa Cruz Biotech, Dallas, TX, USA), anti-cyclin D1 (Santa Cruz Biotech), anti-CDK2 (Santa Cruz Biotech), anti-CDK4 (Santa Cruz Biotech), anti-phospho-PKC*α/β* II(Thr638/641) (Cell Signaling), anti-phospho-PKC*δ/θ*(Ser643/676) (Cell Signaling), anti-phospho-PKC*ζ/λ*(Thr410/403) (Cell Signaling), and anti-actin (Santa Cruz Biotech).

### Annexin V and propidium iodide staining

Apoptosis-mediated cell death was examined using a FITC-annexin V apoptosis detection kit (BD Biosciences, San Diego, CA, USA) according to the manufacturer’s instructions. Briefly, cells were incubated with 5 μl of FITC annexin V, after which 5 µl of propidium iodide (PI) was added. Stained cells were analyzed by flow cytometric analysis.

### Bromodeoxyuridine incorporation assays

Incorporation of bromodeoxyuridine (BrdU) into cellular DNA was measured using a colorimetric enzyme-linked immunosorbent assay kit (Roche Applied Science, Indianapolis, IN, USA) according to the manufacturer’s instructions.

### Senescence associated-*β*-galactosidase staining, colony-forming activities, and cell cycles

These experiments were performed as described.^52^

### Sample labeling and Illumina BeadChip array hybridization

These experiments were performed as described.^35^ Array hybridizations were conducted with four sets of RNA samples from independently cultured cell samples per unit time. Array data processing and analysis was performed using Illumina BeadStudio software (Illumina. Inc., San Diego, CA, USA).

### Statistical analysis

Statistical significance of values between the various experimental groups were determined using Student’s two-tailed *t*-test.
