# Supplementary material for: Acyl-CoA thioesterase 7 is involved in cell cycle progression via regulation of PKCζ–p53–p21 signaling pathway
Source: Cell Death Dis. 2017 May 18;8(5):e2793–. doi: 10.1038/cddis.2017.202 (PMC5584527; doi:10.1038/cddis.2017.202)
Supplement: Supplementary Figures [file cddis2017202x1.ppt]

## Slide 1
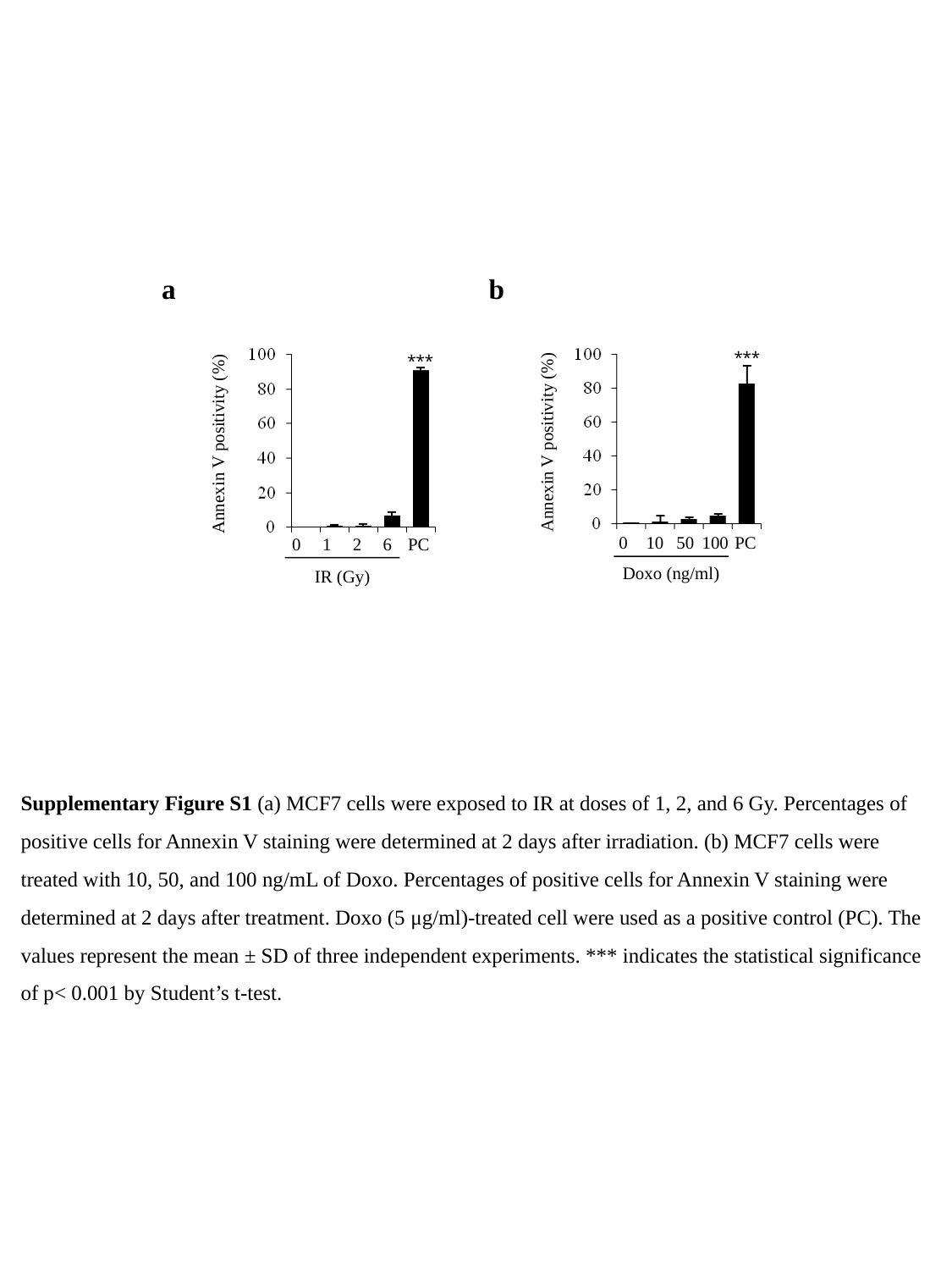

a
b
***
***
Annexin V positivity (%)
Annexin V positivity (%)
0
10
50
100
PC
0
1
2
6
PC
Doxo (ng/ml)
IR (Gy)
Supplementary Figure S1 (a) MCF7 cells were exposed to IR at doses of 1, 2, and 6 Gy. Percentages of positive cells for Annexin V staining were determined at 2 days after irradiation. (b) MCF7 cells were treated with 10, 50, and 100 ng/mL of Doxo. Percentages of positive cells for Annexin V staining were determined at 2 days after treatment. Doxo (5 μg/ml)-treated cell were used as a positive control (PC). The values represent the mean ± SD of three independent experiments. *** indicates the statistical significance of p< 0.001 by Student’s t-test.

## Slide 2
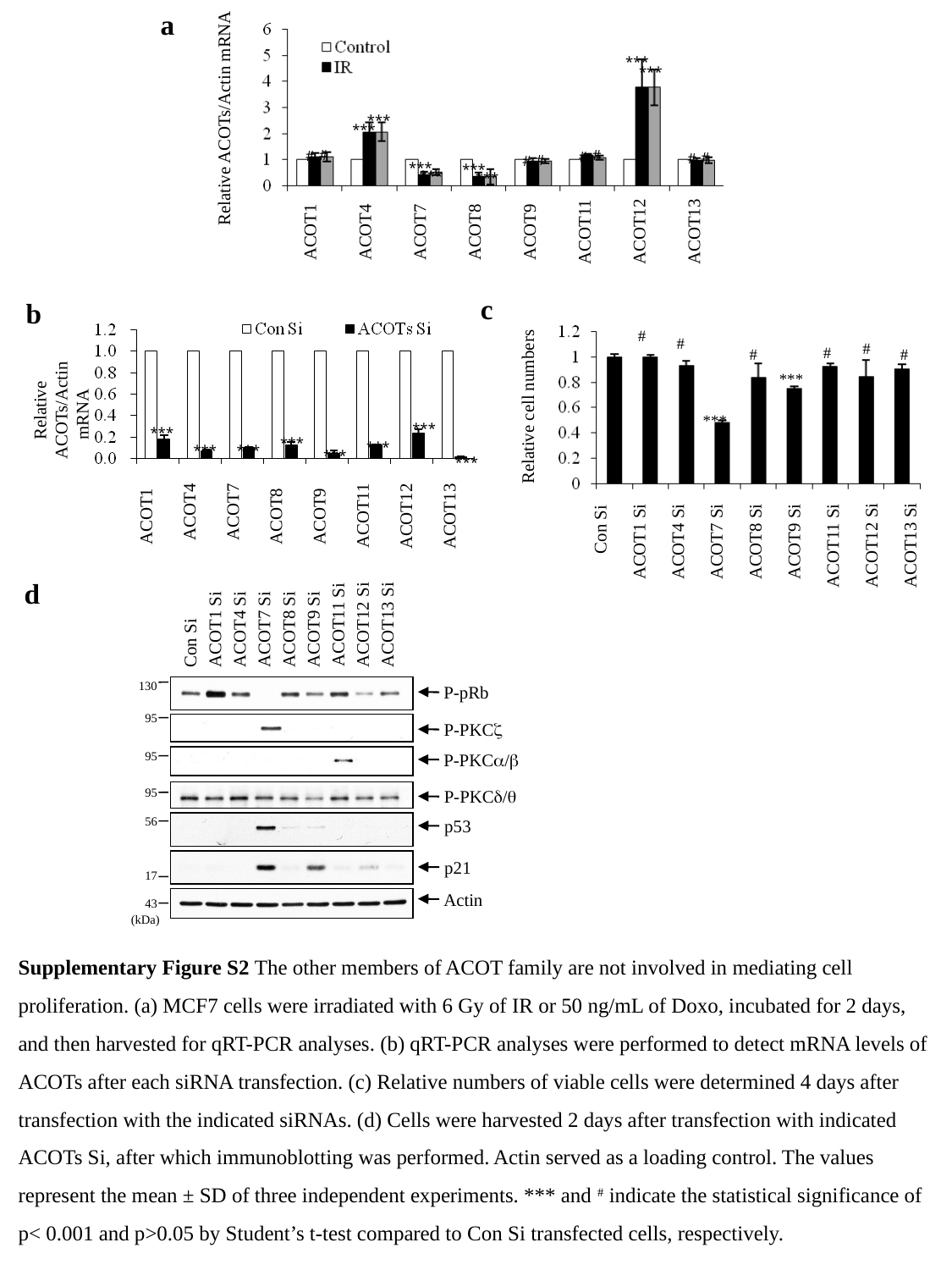

Relative ACOTs/Actin mRNA
ACOT1
ACOT4
ACOT7
ACOT8
ACOT9
ACOT11
ACOT12
ACOT13
a
***
***
***
***
#
#
#
#
#
#
#
#
***
***
***
***
Relative ACOTs/Actin mRNA
ACOT4
ACOT7
ACOT1
ACOT8
ACOT9
ACOT11
ACOT12
ACOT13
***
***
***
***
***
***
***
***
c
b
#
#
#
#
#
#
***
Relative cell numbers
***
Con Si
ACOT1 Si
ACOT4 Si
ACOT7 Si
ACOT8 Si
ACOT9 Si
ACOT11 Si
ACOT12 Si
ACOT13 Si
d
ACOT11 Si
ACOT12 Si
ACOT13 Si
ACOT7 Si
ACOT1 Si
ACOT4 Si
ACOT8 Si
ACOT9 Si
Con Si
130
P-pRb
95
P-PKC
95
P-PKCα/β
95
P-PKCδ/
56
p53
p21
17
Actin
43
(kDa)
Supplementary Figure S2 The other members of ACOT family are not involved in mediating cell proliferation. (a) MCF7 cells were irradiated with 6 Gy of IR or 50 ng/mL of Doxo, incubated for 2 days, and then harvested for qRT-PCR analyses. (b) qRT-PCR analyses were performed to detect mRNA levels of ACOTs after each siRNA transfection. (c) Relative numbers of viable cells were determined 4 days after transfection with the indicated siRNAs. (d) Cells were harvested 2 days after transfection with indicated ACOTs Si, after which immunoblotting was performed. Actin served as a loading control. The values represent the mean ± SD of three independent experiments. *** and # indicate the statistical significance of p< 0.001 and p>0.05 by Student’s t-test compared to Con Si transfected cells, respectively.

## Slide 3
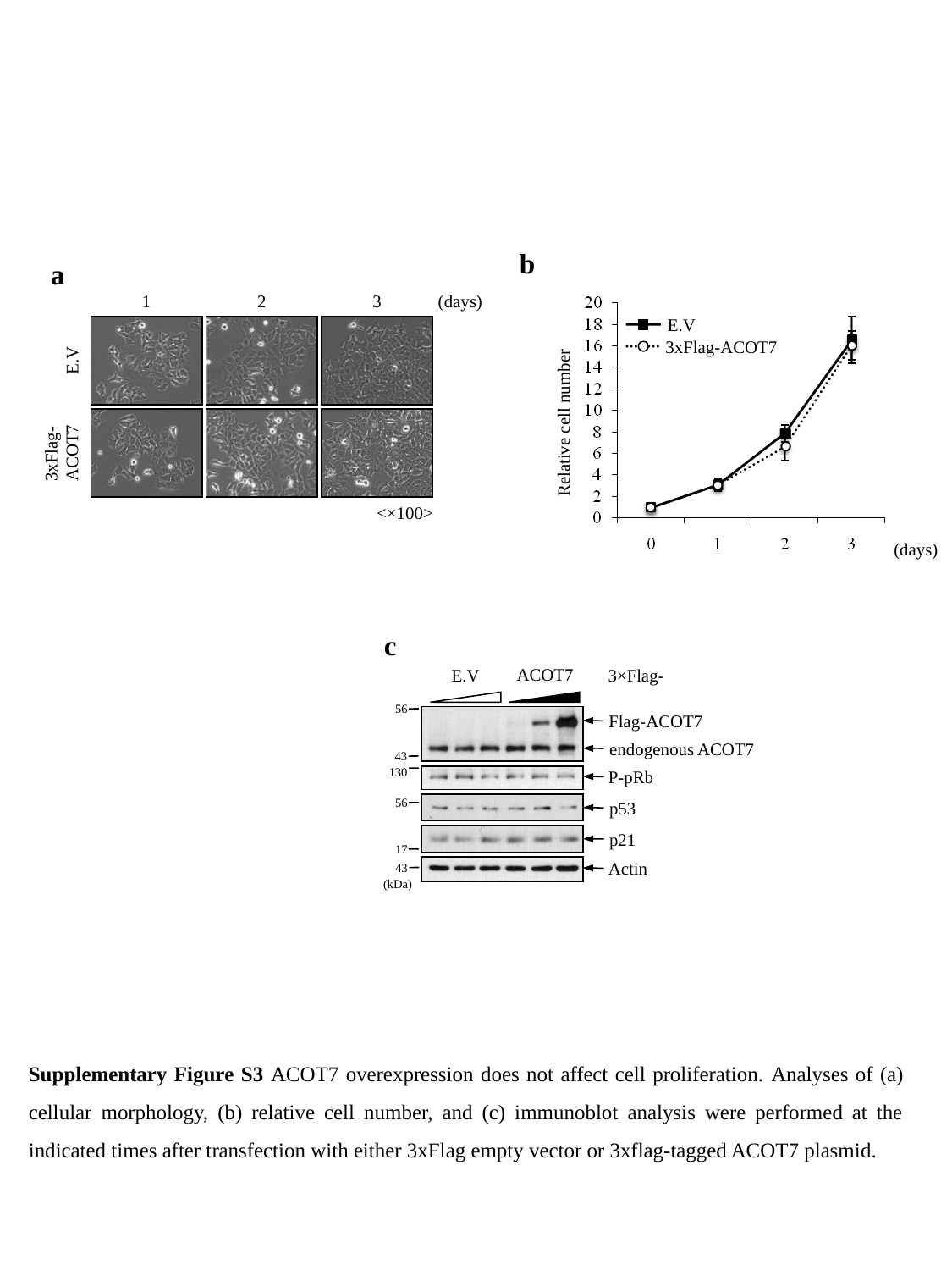

b
a
1
2
3
(days)
E.V
3xFlag-
ACOT7
<×100>
E.V
3xFlag-ACOT7
Relative cell number
(days)
c
ACOT7
E.V
3×Flag-
Flag-ACOT7
endogenous ACOT7
P-pRb
p53
p21
Actin
56
43
130
56
17
43
(kDa)
Supplementary Figure S3 ACOT7 overexpression does not affect cell proliferation. Analyses of (a) cellular morphology, (b) relative cell number, and (c) immunoblot analysis were performed at the indicated times after transfection with either 3xFlag empty vector or 3xflag-tagged ACOT7 plasmid.

## Slide 4
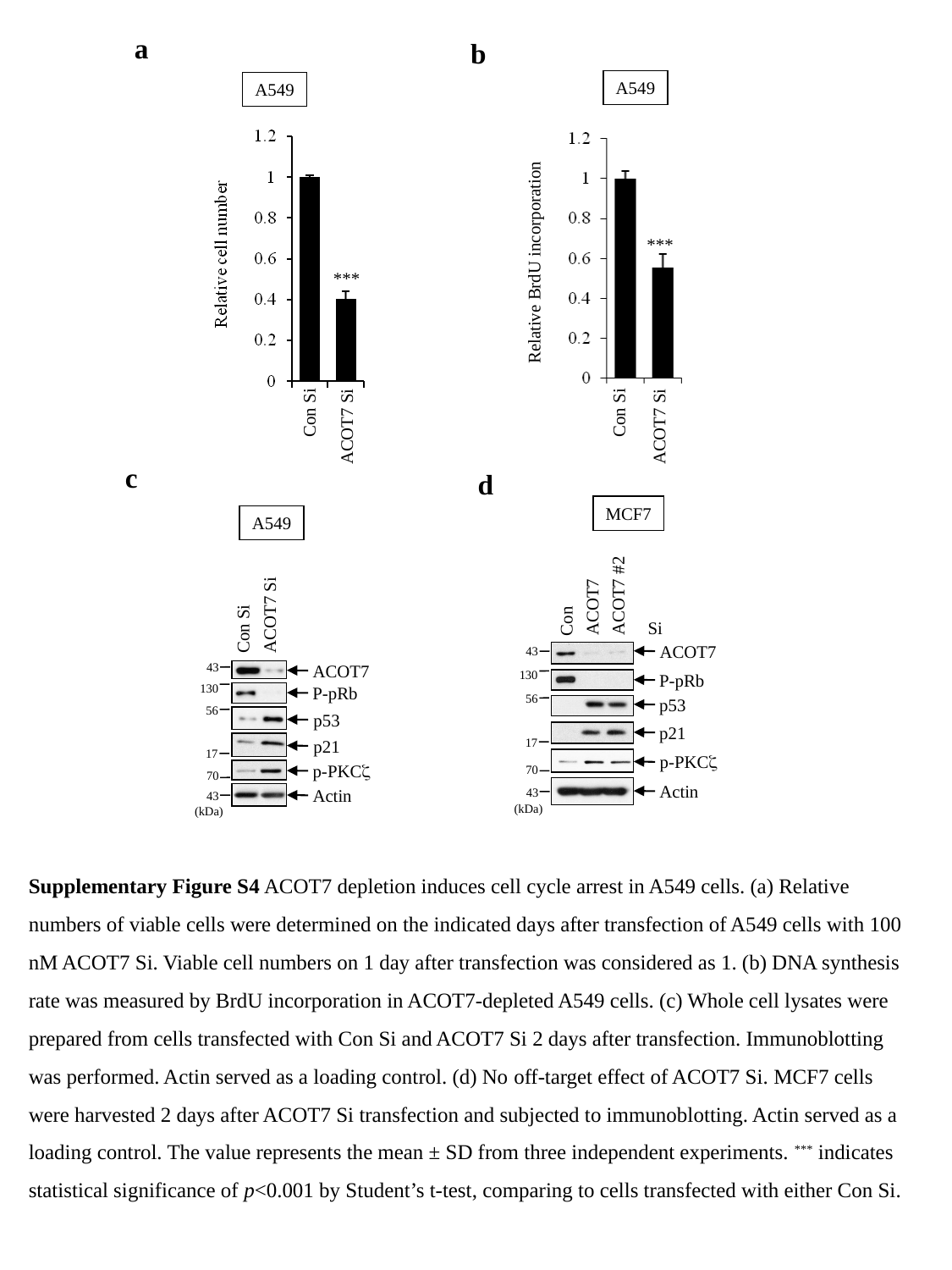

a
b
A549
Relative BrdU incorporation
Con Si
ACOT7 Si
***
A549
Con Si
ACOT7 Si
***
c
d
MCF7
ACOT7 #2
ACOT7
Con
Si
ACOT7
P-pRb
p53
p21
p-PKC
Actin
A549
ACOT7 Si
Con Si
P-pRb
p53
p21
p-PKC
Actin
ACOT7
43
43
130
130
56
56
17
17
70
70
43
43
(kDa)
(kDa)
Supplementary Figure S4 ACOT7 depletion induces cell cycle arrest in A549 cells. (a) Relative numbers of viable cells were determined on the indicated days after transfection of A549 cells with 100 nM ACOT7 Si. Viable cell numbers on 1 day after transfection was considered as 1. (b) DNA synthesis rate was measured by BrdU incorporation in ACOT7-depleted A549 cells. (c) Whole cell lysates were prepared from cells transfected with Con Si and ACOT7 Si 2 days after transfection. Immunoblotting was performed. Actin served as a loading control. (d) No off-target effect of ACOT7 Si. MCF7 cells were harvested 2 days after ACOT7 Si transfection and subjected to immunoblotting. Actin served as a loading control. The value represents the mean ± SD from three independent experiments. *** indicates statistical significance of p<0.001 by Student’s t-test, comparing to cells transfected with either Con Si.

## Slide 5
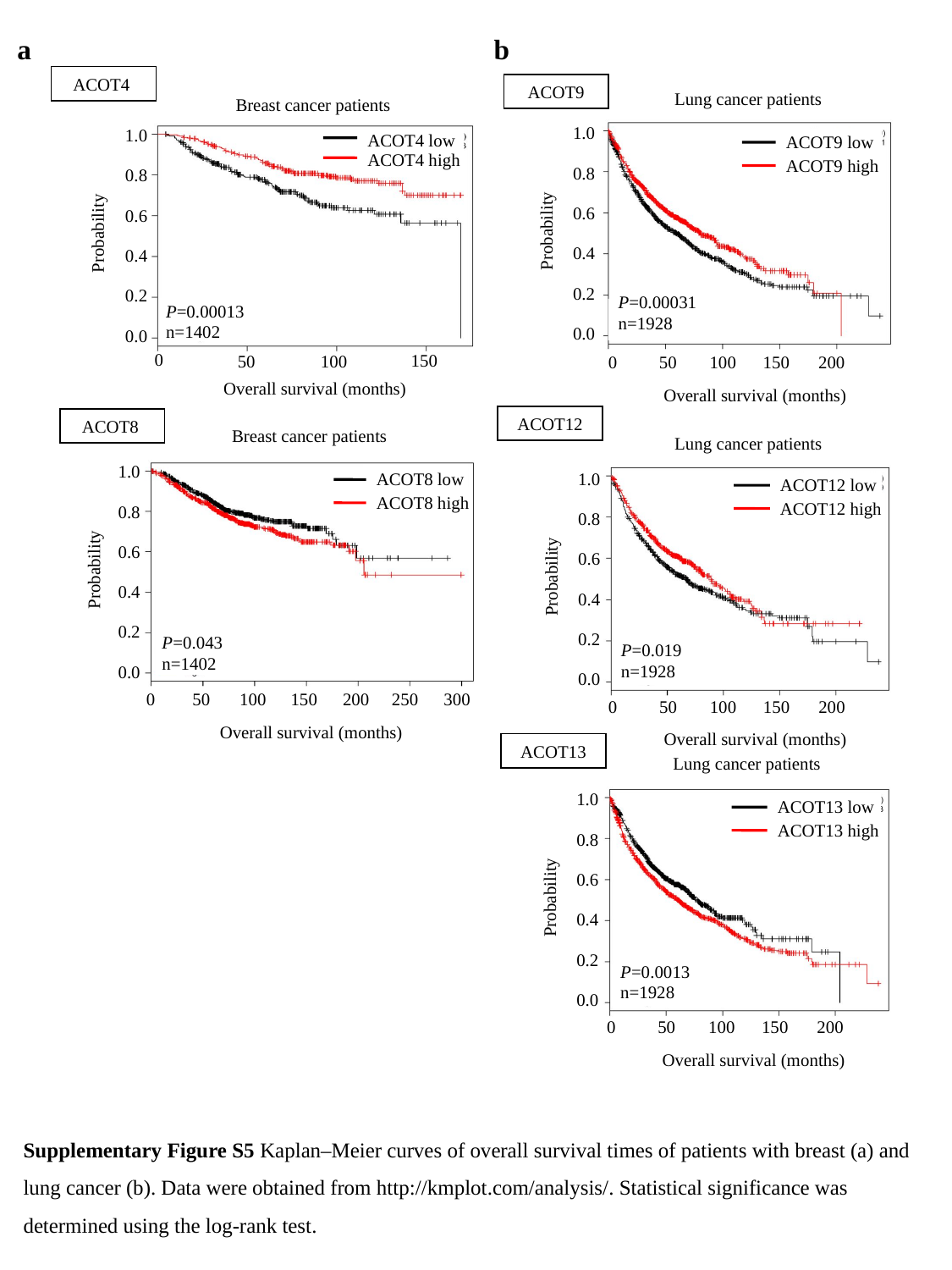

a
b
ACOT4
ACOT9
Lung cancer patients
1.0
ACOT9 low
ACOT9 high
0.8
0.6
Probability
0.4
0.2
P=0.00031
n=1928
0.0
0
50
100
150
200
Overall survival (months)
Breast cancer patients
1.0
ACOT4 low
ACOT4 high
0.8
0.6
Probability
0.4
0.2
P=0.00013
n=1402
0.0
0
150
50
100
Overall survival (months)
ACOT12
ACOT8
Breast cancer patients
1.0
ACOT8 low
ACOT8 high
0.8
0.6
Probability
0.4
0.2
P=0.043
n=1402
0.0
0
50
100
150
200
250
300
Overall survival (months)
Lung cancer patients
1.0
ACOT12 low
ACOT12 high
0.8
0.6
Probability
0.4
0.2
P=0.019
n=1928
0.0
0
50
100
150
200
Overall survival (months)
ACOT13
Lung cancer patients
1.0
ACOT13 low
ACOT13 high
0.8
0.6
Probability
0.4
0.2
P=0.0013
n=1928
0.0
0
50
100
150
200
Overall survival (months)
Supplementary Figure S5 Kaplan–Meier curves of overall survival times of patients with breast (a) and lung cancer (b). Data were obtained from http://kmplot.com/analysis/. Statistical significance was determined using the log-rank test.
